# Potential drug interactions and duplicate prescriptions among ambulatory cancer patients: a prevalence study using an advanced screening method

**DOI:** 10.1186/1471-2407-10-679

**Published:** 2010-12-13

**Authors:** Roelof WF van Leeuwen, Eleonora L Swart, Frits A Boom, Martin S Schuitenmaker, Jacqueline G Hugtenburg

**Affiliations:** 1 Department of Clinical Pharmacology and Pharmacy, VU University medical center, Amsterdam, The Netherlands; 2 Department of Clinical Pharmacy, Zaans Medical Center, Zaandam, The Netherlands; 3 EMGO Institute for Health and Care Research, VU University medical center, Amsterdam, The Netherlands

## Abstract

**Background:**

The pharmacotherapeutic treatment of patients with cancer is generally associated with multiple side-effects. Drug interactions and duplicate prescriptions between anti-cancer drugs or interactions with medication to treat comorbidity can reinforce or intensify side-effects.

The aim of the present study is to gain more insight into the prevalence of drug interactions and duplicate prescriptions among patients being treated in the outpatient day care departments for oncology and hematological illnesses. For the first time the prevalence of drug interactions with OTC-drugs in cancer patients will be studied. Possible risk factors for the occurrence of these drug-related problems will also be studied.

**Methods/Design:**

A multicenter cross-sectional observational study of the epidemiology of drug interactions and duplicate prescriptions is performed among all oncology and hemato-oncology patients treated with systemic anti-cancer drugs at the oncology and hematology outpatient day care department of the VU University medical center and the Zaans Medical Center.

**Discussion:**

In this article the prevalence of potential drug interactions in outpatient day-care patients treated with anti-cancer agents is studied using a novel more extensive screening method. If this study shows a high prevalence of drug interactions clinical pharmacists and oncologists must collaborate to develop a pharmaceutical screening programme, including an automated electronic warning system, to support drug prescribing for ambulatory cancer patient. This programme could minimize the occurrence of drug related problems such as drug interactions and duplicate prescriptions, thereby increasing quality of life.

**Trial registration:**

This study is registered, number NTR2238.

## Background

The pharmacotherapeutic treatment of patients with cancer is generally associated with multiple side-effects. The cause of the side-effects is usually due to the toxicity of the drugs themselves. In addition, drug interactions can intensify side-effects. In general, interactions are the cause of approximately 20-30% of all drug side-effects, of which 70% needs clinical attention and 1-2% is even life-threatening [1]. Cancer patients are particularly susceptible to drug interactions [2]. In addition to chemotherapy, cancer patients often use co-medication to treat cancer related pain and venous thrombosis or to reduce the side-effects of the anti-cancer drugs. Interactions with drugs used to treat comorbidities can also occur.

Drug interactions in nature are subdivided into two types; pharmacokinetic and pharmacodynamic interactions. Pharmacokinetic interactions alter the absorption, distribution, metabolism or excretion of a drugs. The majority of pharmacokinetic interactions are the result of inhibiting the liver enzymes Cytochrome P450 [3]. Many anti-cancer drugs are metabolised via this mechanism [4-6]. Furthermore, in cancer patients the illness itself may also influence the pharmacokinetics of drugs. For example the absorption of drugs can change as a result of malnutrition and mucositis [2]. Impaired kidney and liver function can also result in an abnormal metabolism and excretion of a drug [2]. A pharmacodynamic interaction occurs when two or more drugs act on the same target site of clinical effect. Pharmacodynamic interactions can be additive, synergistic or antagonistic and may influence the effectiveness or side effects of drugs [3].

There is very little data available about the occurrence of interactions and duplicate descriptions in patients being treated with anticancer drugs. In literature various studies can be found that describe the occurrence of interactions and duplicate prescriptions in general clinical departments [7-9]. However, the results of these studies are not representative for cancer patients. Riechelmann et al [2] studied the prevalence of drug interactions and duplicate descriptions between anti-cancer drugs and medication to treat comorbidities. This study showed that 27% of cancer patients are exposed to interactions between anti-cancer drugs and other drugs. Not one study has been undertaken into the effect of using "Over The Counter" medication (OTC medication). The clinical relevance of interactions between anti-cancer drugs and OTC drugs is not entirely clear. It is presumed that these interactions are seriously under-reported [3].

Risk factors for the occurrence of interactions among the general population are described extensively in literature. Research among the general population shows that lung patients, patients who use anti-coagulant drugs, patients with cardiovascular disease who use, amongst other things, diuretics, nitrates, ACE-inhibitors and Ca antagonists, patients older than 50 and patients suffering from diabetes and kidney disorders belong to the group with risk factors for the occurrence of interactions and duplicate prescriptions [10,11]. It is not certain whether these risks factors in cancer patients using anti-cancer drugs are the same. Research by Riechelmann et al. into clinical oncology patients suggests that the use of eight or more drugs and a hospital stay of more than six days represented a risk factor [12]. In ambulatory cancer patients Riechelmann et al found an increased risk for certain types of cancer (mainly brain tumours) and patients receiving drugs to treat comorbidities [2].

Due to the lack of data available to the prescribing oncologist about medication to treat comorbidities of cancer patients, some drug-related problems such as interactions and duplicate prescriptions that can occur during the use of anti-cancer drugs are not being recognised. Furthermore, due to poor transfer of medication information between the community pharmacy, the general practitioner and the hospital, these drug-related problems may not always be identified.

The aim of the present study is to gain more insight into the prevalence of interactions and duplicate prescriptions among patients being treated in the outpatient day care departments for oncology and hemato-oncology using a novel more extensive screening method. For the first time the prevalence of drug interactions with OTC-drugs in cancer patients will be studied. Possible risk factors for the occurrence of these drug-related problems will also be studied.

## Methods

### Study design and setting

A multicenter cross-sectional study of the epidemiology of drug interactions and duplicate prescriptions is performed during a five month period starting in november 2009 among all oncology and hemato-oncology patients treated with systemic anti-cancer drugs at the (hemato-)oncology outpatient day care department of the VU University Medical Center and the Zaans Medical Center. The VU University medical center is a large tertiary referral hospital in Amsterdam. The Zaans Medical Center is a small community hospital situated in Zaandam (Amsterdam area).

### Patients

In a five month period all patients with solid and hematologic malignancy currently using systemic anti-cancer drugs are asked to participate in the study. Exclusion criteria: the use of trial medication, a lack of command of the Dutch language and younger than 18 years old. All participating patients are asked to sign Informed Consent. The study is approved by the Medical Ethics Board of the VU University medical center and the Zaans Medical center.

### Patient interview

Patients are asked questions by means of a structured interview (RVL) (available online as an additional file). Questions concern co-morbidities and the use of OTC drugs. To determine the type of co-medication, an overview of drugs prescribed is obtained from the community pharmacy and the actual use is discussed with the patient. Data on the use of anti-cancer agents, diagnosis, aim of treatment (palliative/adjuvant), treatment start date and cancer-related co-medication is collected by means of a medical chart review and, if necessary, by means of interview of the prescribing doctor. Data on renal function (creatinine) and liver function tests [aspartate aminotransferase (ASAT), alanine aminotransferase (ALAT) and gamma-glutamyltransferase (γ-GT)] is obtained from the laboratory database of the hospital.

We define a laboratory abnormality as an increase of 50% or greater above the upper limit in plasma levels measured within the prior 4 weeks (upper normal limits: ASAT≤35 U/L, ALAT≤40 U/L, γ-GT≤44 U/L, creatinine≤99 μmol/L).

### Determinations of potential drug interactions and duplicate prescriptions

Drugs are subdivided into four groups; "anti-cancer agents", "supportive care agents", "medications to treat comorbidity" and "OTC-drugs". We define "anti-cancer agents" as medication to treat solid or hematological malignancies, "supportive care agents" as medications to treat cancer- and/or therapy- related symptoms, "medications to treat comorbidity" as a non cancer clinical condition that required pharmacologic treatment and "OTC-drugs" as (alternative) medications and food supplements used on the patients own initiative without prior consultation of a doctor. For each patient we add up the number of medications by group. If a medication contained two or more pharmacologically active agents each drug is counted individually in the analysis (e.g. sulfamethoxazol combined with trimethoprim). The drug is counted only once when a patient was taking the same medication on more than one regimen (e.g. long- and short-acting morphine). We define a duplicate prescription as the concurrent use of two drugs of the same class to treat the same condition. A duplicate prescription can be both desirable (e.g. long- and short-acting morphine) and undesirable. In this study only undesirable duplicate prescriptions were analysed.

Drugs are screened for potential drug interaction by the Drug Interaction Facts Software (Facts and Comparisons, version 4.0) [13], which has been shown to have an accuracy of over 95% in detecting drug interactions [14]. The program classifies interactions by level of severity and level of scientific evidence [13]. Classification is described in Table 1. Because of lack of clinical significance drug interactions of minor severity will not be included in the analysis.

**Table 1:** Classification of drug interactions by Drug Interaction Facts Software 13

Classification	Description
Severity	

1	Major: life-threatening or permanent damage

2	Moderate: deterioration of patient's status, treatment is required

3	Minor: bothersome or little effect

Documentation	

1	Established: proven to occur in well-controlled studies

2	Probable: very likely, but not proven clinically

3	Suspected: may occur; some good data, but needs more study

4	Possible: could occur, but data are very limited

5	Unlikely: doubtful; no good evidence of a clinical effect

Additionally, all drugs are manually screened for combinations of drugs with QT-interval prolongating and/or Torsades de Pointes (TdP) inducing properties using the Arizona CERT system [15] and PubMed (QT-interaction). Because of the potentially severe consequences we classify all drug combinations with risk for QT-prolongation as major. The level of scientific evidence is determined by screening the electronic database PubMed.

Furthermore, drugs that may cause increased risk of falling (CNS-depressant agents) are identified by using handbooks and PubMed. If a combinations of two CNS-depressant agents is detected this is counted as one drug interaction (CNS-interaction) in the analyses. We classify all CNS-interactions as moderate. The level of evidence is determined by screening the electronic database PubMed.

Drugs are also manually screened for the combination between NSAID's and corticosteroids, anticoagulants, aspirin or SSRI's (GI-interaction). These combinations are known to increase the gastrointestinal bleeding risks [16,17]. Because of the potentially severe consequences we classify all GI-interactions as major. The level of scientific evidence is determined by screening the electronic database PubMed.

To identify drug interactions of OTC-drugs we screen pharmacology handbooks and electronic databases PubMed and Thomson Micromedex.

The medication screened for drug interactions are the medications of the 4 groups mentioned above; "anti-cancer agents", "supportive care agents", "medications to treat comorbidity" and "OTC-drugs". We only count the drug interactions in the analysis when an "anti-cancer drug" or a "supportive care agents" is involved. We don't include potential drug interactions between non-cancer drugs (drug interactions between two medications to treat comorbidity or OTC-drugs) in the analysis.

### Statistical analysis

Only few studies of drug interactions in oncology are published in the literature. A sample of 300 patients is chosen as a feasible sample. Patients will be asked to participate in this study until this number is obtained. Descriptive statistics (means ± SD or median) and frequency analysis is applied to describe the whole study sample with regard to demographics, cancer type, treatment objective, type of anticancer drugs, comorbidities, number of medications per patient and laboratory abnormalities and drug interactions characteristics (severity, level of scientific evidence, onset and mechanism).

Univariate and multivariate logistic regression analysis will be performed to identify potential risk factors for the occurrence of drug interactions. The dependent variable is the number of drug interactions per patient. Expected covariates are age, number of drugs per patient, multicenter (Zaans Medical Center, VU University Medical Center), treatment intent (palliative/curative), treatment type (e.g. chemotherapy, hormone therapy, combination therapy), presence of comorbidities, tumor type (hemato-oncology/oncology) and the use of at least one OTC-drug. Gender is not included as a covariate due to the fact that certain cancer types only occur in men or women. For binary or nominal variables, the largest group is taken as the referent. Variables with univariate P-values <0.1 are included in the multivariate analysis. In the multivariate analysis a P-value of <0.05 is considered statistically significant. The data are adjusted for possible confounders and effect modifiers (age, sex, type of cancer). Data are collected and analysed in SPSS version 15.0 (SPSS Inc., Chicago, IL 60606).

### Power calculation

The occurrence of drug interactions is the primary endpoint. Literature show that drug interactions occur in approximately 27% of ambulatory cancer patients [2]. With an accuracy of 5% and 95% confidence interval, a total of 300 patients is needed.

## Discussion

In this study the prevalence of potential drug interactions in outpatient day-care patients treated with anti-cancer agents will be investigated using a novel more extensive screening method. Additionally, risk factors for the occurrence of drug interaction will be studied. This study will provide more insights into drug interactions among cancer patients and can be used to improve the pharmaceutical patient care by setting up a routine screening method to identify drug interactions.

The Drug Interaction Facts Software, which is used in this study, shows great sensitivity in identifying drug interactions [14] but doesn't recognise all QT-interactions, interactions that cause gastroduodenal toxicity and CNS-interactions. In this study we will conduct an additional manual search and use other databases to gain more insight in the occurrence of QT-interactions, interactions that can cause gastrointestinal toxicity and CNS-interactions. Numerous drugs, representing a wide range of pharmacologic classes, have been associated with QT interval prolongation [15]. Due to possibly serious and even fatal consequences of drug combinations that cause prolongation of the QT interval has led to contraindicating the use of many drug combinations. Falling in elderly patients is a major public health concern as well. Most falls are the result of a combination of intrinsic and extrinsic risk factors [18]. Prescribed CNS-depressant medication is an important contributor to the risk of falling in elderly [19]. Several commonly used drugs (e.g. psychotropic and cardiovascular drugs) are indentified as a risk factor for falls [20-22]. Multiple CNS-depressant drug use may even lead to increased risk of falling [23]. NSAID's are also used extensively in oncology as an analgesic. However, their use is limited by gastrointestinal toxicity. Additional pharmacological risk factors for the development of NSAID ulcers include concomitant use of corticosteroids, anticoagulants, aspirin and serotonin reuptake inhibitors (SSRI's) [17,18]. The additional search in these databases is expected to increased the number of detected drug interactions in our study population and give a more valid insight in the prevalence of drug interactions in cancer patients.

In this study we only include a drug interaction in the analysis if an anti-cancer or a supportive care agent is involved. Drug interactions between non-cancer agents is beyond the scope of this study. A strength of our study is that we included the use of OTC drugs in our analysis to gain more insight into the prevalence of interactions between OTC-drugs and anti-cancer drugs. It is expected that OTC-drugs are used extensively in cancer patients and the prevalence of drug interactions with OTC-drugs will be high.

Due to the retrospective design of the study the clinical consequences cannot be studied. However, a prospective design could influenced prescribing by oncologist. If this study shows a high prevalence of drug interactions clinical pharmacists and oncologists must collaborate to set up a screening programme to reduce the occurrence of drug related problems. This screening can be implemented for ambulatory cancer patients before using anti-cancer drugs, in order to improve their quality of life and minimize the occurrence of drug interactions.

## Competing interests

The authors declare that they have no competing interests.

## Authors' contributions

RVL, JH and NS developed the idea for this study. RVL was responsible for drafting the manuscript with contributions from JH, NS, FB and MS. RVL will implement the protocol and collect the data. All authors read and approved the final manuscript.

## Pre-publication history

The pre-publication history for this paper can be accessed here:

http://www.biomedcentral.com/1471-2407/10/679/prepub

## Supplementary Material

Additional file 1 **structured interview**. structured interview for collecting data. Click here for file
