# Supplementary material for: Potential drug interactions and duplicate prescriptions among ambulatory cancer patients: a prevalence study using an advanced screening method
Source: BMC Cancer. 2010 Dec 13;10:679. doi: 10.1186/1471-2407-10-679 (PMC3013087; doi:10.1186/1471-2407-10-679)
Supplement: Additional file 1 — structured interview. structured interview for collecting data. [file 1471-2407-10-679-S1.PDF]

Interview

Potential drug interactions and duplicate prescriptions among ambulatory cancer patients

a prevalence study using an advanced screening method

zaans medisch centrum

VU medisch centrum

Part 1: Patient

Date of inclusion: ………..-………..-………..

Date of birth: ………..-………..-………..

Gender: M / W

Part 2: Co-morbidity

1. Health related problems?

	Yes	No	
Diabetes	( )*	( )	**Type: 1 / 2*
Liver disease	( )*	( )	
Cardiovascular disease	( )*	( )	
Kidney disease	( )*	( )	
Lung disease	( )*	( )	
Arthritis	( )*	( )	
TIA/CVA	( )*	( )	
Others*	( )*	( )	

*** Others:***

___

___

___

2. Medication to treat co-morbidity? (besides anticancer therapy)

	Name	***Dose***
1		
2		
3		
4		
5		
6		
7		
8		
9		
10		
11		
12		
13		
14		
15		

Part 3: OTC-medication

OTC-drugs?

	*Yes**	*No*	**Dose?*
Paracetamol	( )	( )	____________
Ibuprofen	( )	( )	____________
Naproxen	( )	( )	____________
Vitamin C	( )	( )	____________
St. John's wort	( )	( )	____________
Ginkgo-Biloba	( )	( )	____________
Zantac	( )	( )	____________
Gaviscon	( )	( )	____________
Others*	( )	( )	

** Other OTC drugs:*

____

____

____

Part 4: Cancer type

Cancer type?

Oncology

Brain	( )	Liver	( )
Breast	( )	Genitourinary	( )
Kidney	( )	Gastrointestinal	( )
Gynaecological	( )	Lung	( )
Melanoma	( )	Other: ________________	( )

Haemato-oncology

Leukaemia	( )	Other: ________________	( )
Myelodysplastic syndrome	( )		
Myeloproliferatic syndrome	( )		
Malignant lymphoma	( )		
Plasma cell dyscrasia	( )		
Immunocytoma	( )		

Part 5: Cancer treatment

1. Start anti-cancer treatment?

Month: ___________________________ **Year:** __________________________

2. Center? Department?

Center:	***Zaans Medical Center / VU Medical Center***
Department:	***Oncology / Haemato-oncology***

3. Treatment type?

	Yes	***No***
Chemotherapy	***( )***	***( )***
Radiotherapy	***( )***	***( )***
Hormonetherapy	***( )***	***( )***
MAB's	***( )***	***( )***
NIB's	***( )***	***( )***
Others*	***( )***	***( )***
Combinations*	***( )***	***( )***

****Others?:***

____

4. Treatment intent?

Curative/Adjuvant	***( )***
Palliative	***( )***

5. Anticancer agents and supportive care drugs?

	Name	*Dose*
1		
2		
3		
4		
5		
6		
7		
8		
9		
10		
11		
12		
13		
14		
15		

Part 6: Laboratory values

Creatinine:	_______	*μmol/l*	→ d.d. ……../………./……..
ALAT:	_______	*U/L*	→ d.d. ……../………./……..
ASAT:	_______	*U/L*	→ d.d. ……../………./……..
γ-GT:	_______	*U/L*	→ d.d. ……../………./……..
